# Silencing of Euchromatic Transposable Elements as a Consequence of Nuclear Lamina Dysfunction

**DOI:** 10.3390/cells9030625

**Published:** 2020-03-05

**Authors:** Valeria Cavaliere, Giovanna Lattanzi, Davide Andrenacci

**Affiliations:** 1 Dipartimento di Farmacia e Biotecnologie, Alma Mater Studiorum Università di Bologna, 40126 Bologna, Italy; valeria.cavaliere@unibo.it; 2 CNR Institute of Molecular Genetics “Luigi-Luca Cavalli-Sforza”, Unit of Bologna, 40136 Bologna, Italy; giovanna.lattanzi@cnr.it; 3 IRCCS Istituto Ortopedico Rizzoli, 40136 Bologna, Italy

**Keywords:** nuclear lamins, nuclear envelope, transposons, TE silencing, gene expression, *LamDm0*, cosuppression

## Abstract

Transposable elements (TEs) are mobile genomic sequences that are normally repressed to avoid proliferation and genome instability. Gene silencing mechanisms repress TEs by RNA degradation or heterochromatin formation. Heterochromatin maintenance is therefore important to keep TEs silent. Loss of heterochromatic domains has been linked to lamin mutations, which have also been associated with derepression of TEs. In fact, lamins are structural components of the nuclear lamina (NL), which is considered a pivotal structure in the maintenance of heterochromatin domains at the nuclear periphery in a silent state. Here, we show that a lethal phenotype associated with *Lamin* loss-of-function mutations is influenced by *Drosophila gypsy* retrotransposons located in euchromatic regions, suggesting that NL dysfunction has also effects on active TEs located in euchromatic loci. In fact, expression analysis of different long terminal repeat (LTR) retrotransposons and of one non-LTR retrotransposon located near active genes shows that *Lamin* inactivation determines the silencing of euchromatic TEs. Furthermore, we show that the silencing effect on euchromatic TEs spreads to the neighboring genomic regions, with a repressive effect on nearby genes. We propose that NL dysfunction may have opposed regulatory effects on TEs that depend on their localization in active or repressed regions of the genome.

## 1. Introduction

Transposable elements (TEs), also called mobile elements, are DNA sequences that can increase their copy number in the host genome inserting into new locations. For this reason, active TEs can be mutagenic and cause genomic instability. According to the mechanism of transposition, TEs can be divided into two major classes: DNA transposons and retrotransposons. DNA transposons transpose via a mechanism of cut-and-paste transposition, while retrotransposons transpose via reverse transcription of an RNA intermediate [1]. Retrotransposons are, in turn, divided into long terminal repeat (LTR) retrotransposon and non-LTR retrotransposons [2,3]. LTR retrotransposons have direct repeats of few hundred base pairs at each end, with structural similarity to retroviruses. Non-LTR retrotransposons, such as *LINE* and *SINE* elements, do not have LTRs or similarity with retroviruses. To counteract the deleterious effects of TEs, host cells have evolved strategies to suppress their activation and mobilization. Mechanisms for suppressing TE mobilization are mainly based on RNA silencing and are highly conserved in eukaryotes [4]. The epigenetic silencing of TEs utilizes small non-coding RNAs (sncRNAs) to degrade cytoplasmic RNA by posttranscriptional gene silencing (PTGS) or to repress transcription by transcriptional gene silencing (TGS). TGS is achieved by formation of heterochromatin [5], and reduction of heterochromatin results in the activation of the transcriptionally repressed regions. A global loss of heterochromatin has been described during aging and is related to the derepression of TEs [6]. For this reason, it has been hypothesized that age-associated reduction of heterochromatin determines the loss of inactivation of TE expression and the consequent mobilization. It is known that heterochromatic regions tend to be associated with the nuclear lamina (NL), a structural scaffold lining the surface of the inner nuclear membrane [7]. NL is constituted by a network of intermediate filaments and has different functions including the chromatin anchorage [8]. Lamins are the major constituents of the NL and are classified into A- and B-type lamins [9]. In humans, the A-type lamins (i.e., lamin A and lamin C) are encoded by the *LMNA* gene, while the B-type lamins (i.e., lamin B1 and lamin B2) are encoded by the *LMNB1* and *LMNB2* genes, respectively. Lamin A and lamin C are produced by alternative splicing of a common primary transcript. In humans, mutations in *LMNA* or other nuclear lamina/envelope genes are responsible for a plethora of diseases termed laminopathies, which include muscular dystrophies, cardiomyopathy, lipodystrophies, and progeroid syndromes [10,11,12,13,14]. Two lamin genes are present in *Drosophila*, even if they cannot be classified in A-type or B-type lamins on the base of the homology sequence [15,16,17,18,19]. One of them, called *Lamin* (*Lam*) or *Lamin Dm0* (*LamDm0*)*,* has been considered the homolog of the vertebrate B-type lamin on the basis of its ubiquitous expression, while the other, called *Lamin C* (*LamC*), has been considered the homolog of vertebrate A-type lamin due to its developmentally regulated expression [20]. However, since *Drosophila* and vertebrate lamins are not orthologous and the expression pattern of the lamins in *Drosophila* and vertebrate evolved independently, it is possible that lamin functions can be performed by genes that have a different expression pattern in flies and mammals [21]. In fact, *Drosophila Lam* mutants show larval locomotion and muscular defects, inability to fly, and walking impairment in adults, which resemble aged wild-type flies [21,22]. These phenotypes are reminiscent to those observed in the nuclear laminopathies (i.e., Emery–Dreifuss muscular dystrophy), which are a group of rare disorders caused by mutations in genes encoding proteins of the nuclear lamina [23]. Mutations in the *Drosophila Lam* gene also cause lethality at different stages of development, with few individuals surviving to adulthood [22,24]. Furthermore, *Lam* mutant flies are sterile, and females have ovaries with gross morphological defects, while the eye shows nuclear migration defects and accumulation of red pigmented material [24]. More recently, activation of a number of retrotransposons has been described in *Lam* mutant larvae and in the fat body of adults, suggesting the involvement of the NL in the repression of TEs [25]. Activation of retrotransposons has also been confirmed in human cells, where silencing of *LMNA* induces a significant increase in the expression of the retrotransposon *LINE-1* [26].

In the present study, we show that the penetrance of a *Lam* lethal phenotype manifesting during the adult eclosion of *Drosophila* [22] is increased by new insertions in euchromatic regions of a retrotransposon named *gypsy*. *Gypsy* is a well characterized LTR retrotransposon of *Drosophila* with three open reading frames, one of which encodes the retrotransposase [2]. This retrotransposon is active in the somatic tissues of the female gonads and integrates in the genome of the progeny, after the transfer to the oocytes [27]. *Gypsy* has been found expressed also in other somatic tissues, like adult heads and fat bodies [25,28]. We demonstrated that *Lam* loss-of-function mutations have a silencing effect on a *gypsy* element located in the *cut* (*ct*) locus. By the analysis of three other LTR retrotransposons, *ZAM*, *Idefix*, and *412*, and one non-LTR retrotransposon named *I-element*, we observed expression silencing when these TEs are located near euchromatic genes. Furthermore, we found that repression of euchromatic TEs also affects the genes located in the neighborhood of their insertion sites, suggesting a spreading of the silencing effect. We also show that the expression pattern of a number of TEs changes considerably in *Lam* mutants with a different genetic background. These observations support the hypothesis that repression or derepression of a specific TE depends on its genomic localization.

## 2. Materials and Methods

### 2.1. Drosophila Stocks

*Drosophila* stocks were maintained on standard cornmeal/yeast medium under 12:12 h light/dark cycle at 25 °C. Canton S has been used as wild-type strains. *Lam^4643^*, *lam^K2^*, and *v^1^* were obtained from Bloomington Stock Center. *w^IR6RevI^* and *w^IR6RevII7^* were kindly provided by Chantal Vaury. *Lam* loss-of-function mutant carrying the *Df(1)l11* deficiency and the *gypsy-lacZ* transgene were obtained by crosses starting from the *Df(1)l11/FM7c/flam^FM7c^; P{gypsy-lacZ.p12}*, and the *Lam^4643^/CyO* lines. *Df(1)l11/FM7c/flam^FM7c^; Lam^4643^/CyO; P{gypsy-lacZ.p12}* flies were then crossed with the *w flam^A^; Lam^K2^/CyO* flies, selecting *Df(1)l11/w flam^A^; Lam^4643^/lam^K2^; P{gypsy-lacZ.p12/+* female for the experiments. *Lam^4643^* and *lam^K2^* alleles were placed in the different genetic backgrounds, crossing two times *Lam^4643^/CyO* or *lam^K2^/CyO* males with *CyO/Sp* females carrying the X chromosome from Canton S; *w, flam^A^*; *w^IR6RevI^*; *w^IR6RevII7^*; *v^1^*. *CyO* flies were selected and crossed to establish the new lines.

### 2.2. Mortality Determination

To calculate mortality during adult ecdysis, enclosed adults and adults that died within the puparium were counted, and percentage was calculated. At least 100 individuals were counted per experiment, and three separate experiments were carried out. Average and SD were calculated. *P* value was calculated using the two tailed unpaired Student’s *t*-test (* *P* < 0.05).

### 2.3. β-Galactosidase Staining

Ovaries were dissected from 3–4-day-old females in PBS then transferred and fixed in PBS plus Triton X-100 (PBT) (PBS with 0.1% Triton X-100) containing 0.1% glutaraldehyde for 5 min and rinsed three times with PBT. Each sample was incubated in a 0.2% X-gal staining solution (10 mM phosphate buffer, pH 7.2, 1 mM MgCl2, 5 mM K4[FeII(CN)6], 5 mM K3[Fe(III)(CN)6], 0.1% Triton X-100) for 45 min. After staining, each sample was rinsed three times with PBT, mounted in PBS containing 50% glycerol, and analyzed by bright-field microscopy on a Nikon Eclipse 90i microscope equipped with a 12V, 100W halogen lamp by using a Nikon CFI60 (Chromatic Aberration Free Infinity) Plan Fluor 20x objective with numerical aperture 0.50 and Nomarski optics. Digital images were acquired with a Nikon Digital Sight camera and assembled using the Adobe Photoshop software. No biased image manipulations were applied.

### 2.4. Quantitative RT-PCR

Total RNA was extracted by crushing about 20 heads, 10 ovaries, or whole adult in TRI Reagent (Sigma-Aldrich). Each sample was prepared from 0- to 24-h-old females. Samples were then treated with TURBO DNase (Ambion), and complementary DNAs (cDNAs) were prepared using the High-Capacity RNA-to-cDNA Kit (Thermo Fisher Scientific) or M-MLV Reverse Transcriptase (Ambion) with specific primers, according to the manufacturer’s protocol. When strand-specific expression was analyzed, cDNAs were produced using strand-specific primers (see Table S1). Gene expression was quantified by qPCR using Power SYBR Green PCR master mix (Applied Biosystems) and analyzed by StepOnePlus Real-Time PCR System (Applied Biosystems). Dissociation curve analysis was then performed to determine target specificity. Two or three technical replicates were used for each experimental sample. Transcript levels were normalized to the internal standard gene *Rp49*. To measure the fold change of expression levels between experiments and their specific controls, the ΔΔCt method was used. Three biological replicates were used, and the average and SD were calculated. Differences between experiments and controls were tested with the Student’s *t*-test (* *P* < 0.05, ** *P* < 0.01, *** *P* < 0.005). For the qPCR primer list, see Table S1.

### 2.5. Microscopy Analysis of Adult Eyes

Whole adult eyes were photographed with the Nikon Digital Sight camera mounted on a Nikon Eclipse 90i microscope equipped with a 12V, 100W halogen lamp by using a Nikon CFI60 (Chromatic Aberration Free Infinity) Plan Fluor 10x objective with numerical aperture 0.30. Z-stacks of adult eye images were flattened by using the NIS-Elements Imaging Software. Digital images were assembled using the Adobe Photoshop software. No biased image manipulations were applied.

## 3. Results

### 3.1. Pharate Mortality Induced by Lam Loss-of-Function Mutations is Linked to Gypsy Retrotransposon Silencing

**Figure 1:**
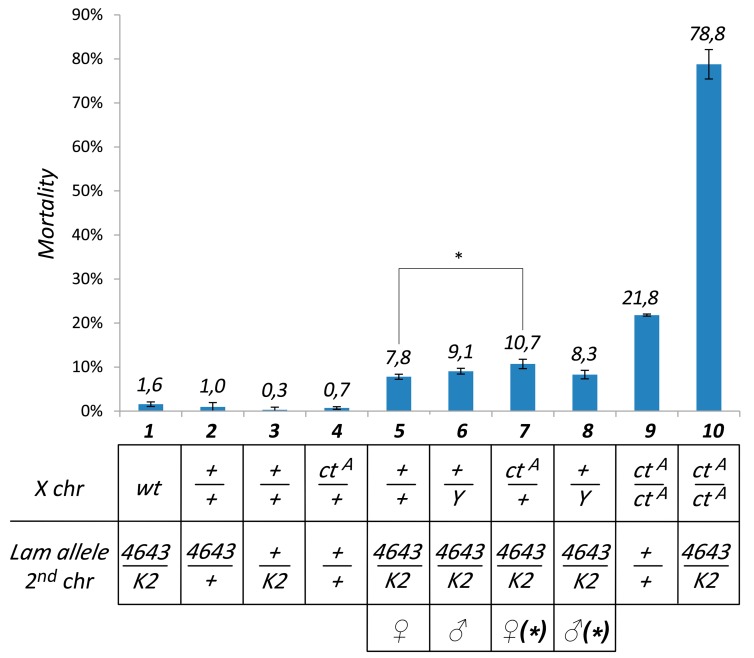
Mutation of the *Lam* gene induces mortality during adult eclosion, which is increased by a *gypsy* insertion in the euchromatic *cut* locus. Mortality during adult eclosion of pharate with different genotypes. Column 1: flies with X chromosomes from the wild type *Canton S* (wt) strain. Columns 2–10: flies with X chromosomes carrying the *flamenco* permissive allele *flam^A^*, which allows *gypsy* activation. These flies can have the *gypsy* induced mutation (*ct^A^*) in heterozygosis, in homozygosis/hemizygosis, or the wild type allele (+). Flies can be homozygous for the wild type *Lam* allele (+/+), heterozygous for one of the two loss-of-function *Lam* alleles (4643/+ or +/K2) or transheterozygous (4643/K2). Y: Y chromosome. Asterisk: females and males derived from the same genetic cross. Data are mean values from three independent experiments, and error bars indicate SD (* *P* < 0.05).

**Figure 2:**
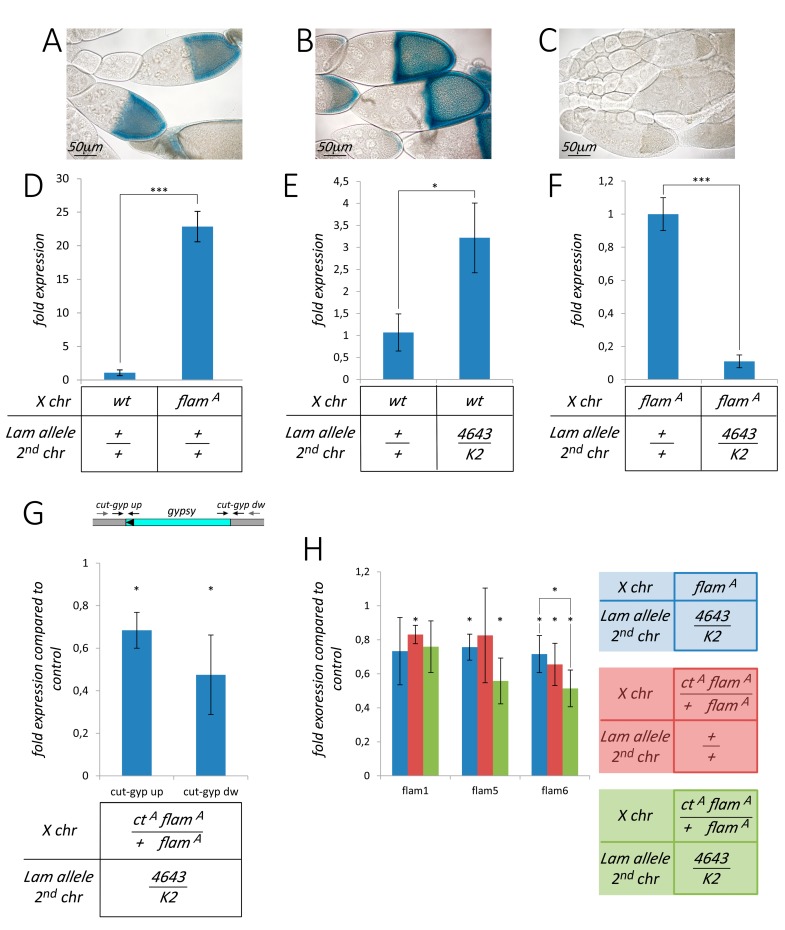
*Lam* inactivation determines the silencing of *gypsy* sequences when *gypsy* is active. (**A**–**C**) Representative egg chambers subjected to β-galactosidase staining as readout for *gypsy-lacZ* reporter activity. (**A**) *gypsy-lacZ* reporter activity is derepressed when the X chromosome from wild type *Canton S* flies is combined with the *Df(1)l11* deficiency encompassing the *flamenco* locus. (**B**) Derepression of the *gypsy-lacZ* reporter activity in flies with the X chromosome carrying the permissive *flam^A^* allele in combination with the *Df(1)l11* deficiency (*Df(1)l11*/*flam^A^*). (**C**) *gypsy-lacZ* reporter activity of *Df(1)l11*/*flam^A^* gonads is repressed in the *Lam^4643/K2^* genetic background. (**D**–**H**) Gene expression analysis of *gypsy* and *flamenco* sequences in RNAs isolated from female head tissues. wt: X chromosomes from the wild type *Canton S* strain. *flam^A^*: X chromosomes carrying the *flamenco* permissive allele *flam^A^*. *ct^A^*: presence of a *gypsy*-induced mutation in the *cut* locus. Flies can be homozygous for the wild type *Lam* allele (+/+) or transheterozygous (4643/K2). Data are mean values from three independent experiments, and error bars indicate SD (* *P* < 0.05; *** *P* < 0.005) (**D**) *gypsy* expression levels in wild type *Canton S* (wt) and *flam^A^* female head tissues. (**E**) *gypsy* expression levels in control and *Lam^4643/K2^* female head tissues carrying X chromosomes from the wild type *Canton S* (wt) strain. (**F**) *gypsy* expression levels in control and *Lam^4643/K2^* female head tissues carrying X chromosomes from the *flam^A^* strain. (**G**) Upper part: schematic representation of the genomic region containing the 5′ upper region of the *cut* gene (grey) where the *gypsy* element (cyan) is inserted. The two strand-specific primers used for RT experiments are represented by grey arrows, while the two qPCR couples of primers are represented by black arrows (see Table S1). Lower part: strand-specific qRT-PCR analysis of the transcription levels of the boundaries of the *gypsy* elements and the surrounding genomic regions of the *cut* locus (*ct^A^* allele) in *ct^A^ flam^A^/flam^A^; Lam^4643/K2^* female head tissues compared to the *ct^A^ flam^A^/flam^A^; Lam^+^* control. ct-gyp up is the upstream boundary, and ct-gyp dw is the downstream boundary. (**H**) qRT-PCR analysis of three *flamenco* fragments in head tissues of *flam^A^* females with or without a copy of the *ct^A^* allele and carrying wild type or *Lam* transheterozygous combination compared to the *flam^A^; Lam^+^* control. flam1 sequence was selected inside the *flamenco* locus in a region without homology with *gypsy*, while flam5 and flam6 were selected inside *gypsy* fragments [31].

Mutations in the *Lam* gene induce mortality in different stages of the *Drosophila* development such as during the pharate adult stage [24]. To study the effect of the *Lam* gene inactivation, we used a transheterozygous combination of two loss-of-function alleles: *Lam^4643^* and *Lam^K2^* (Table S2). The *Lam^4643^* allele is produced by the insertion of a P-element in the first intron of the gene, 258 bp upstream of the translation start site [29], while *Lam^K2^* is produced by a frameshift mutation after amino acid 153 [30]. We analyzed the mortality rate in *Lam^4643/K2^* pharate, finding that the combination of these two loss-of-function alleles does not induce significant pharate mortality (Figure 1, column 1). However, we considered the possibility that this phenotype could depend on the genetic background. In a previous report, we showed that pharate mortality during the eclosion process can be induced by euchromatic insertions of the *gypsy* retrotransposon in genetic backgrounds that are permissive for *gypsy* transposition [31]. To test a possible involvement of *gypsy*, the X chromosomes of the original *Lam^4643/K2^* flies, derived from the wild type *Canton S* strain, were replaced with the X chromosome carrying *flam^A^*, a permissive allele of *flamenco*. The locus *flamenco* is a well characterized sncRNA cluster controlling *gypsy* and other retrotransposons [31]. In this genetic background, *Lam* inactivation induces a low but significant mortality rate during the pharate stage (Figure 1, columns 5 and 6). However, in spite of the difference in the mortality rate, we found that also Canton S flies carry a *flamenco* allele that allows *gypsy* mobilization (Figure 2A,B). This finding indicated that the presence of *flamenco* permissive alleles per se is not the cause of the increased mortality levels. Expression analysis revealed an about 20-fold higher *gypsy* expression level in the heads of adult females carrying the X chromosome with the *flam^A^* allele compared to the flies carrying the X chromosomes of *Canton S* (Figure 2D). This suggested the presence of a higher number of active *gypsy* elements in the *flam^A^* genetic background. To confirm that pharate mortality was linked to the presence of active *gypsy* elements, we tested the effect of the addition of *gypsy* insertions on the mortality rate induced by *Lam* mutations. With this aim, we analyzed the mortality rate of *Lam^4643/K2^* mutant flies carrying a *gypsy* insertion in the *cut* locus (*ct^A^* allele). This *gypsy* insertion was isolated among *flam^A^* individuals, and the resulting mutant has the same genetic background as the original *flam^A^* strain [31]. We found that the *ct^A^* allele in heterozygous condition slightly but significantly increases mortality (Figure 1, compare column 5 with 7), while in homozygous condition it greatly increases the mortality rate of the pharate (Figure 1, column 10). In addition, we tested a second *gypsy* insertion isolated in the *flam^A^* genetic background but in a different locus [31], obtaining a significant increase in pharate mortality when the mutation was in hemizygosis or homozygosis (Figure S1A, columns 4 and 5). These data suggest that mortality of pharate adults found in *Lam* mutant flies is linked to the presence of active *gypsy* elements in euchromatic loci. We expected that the about 20-fold higher *gypsy* expression level found in the *flam^A^* genetic background compared to that of Canton S (Figure 2D) could be due to the fact that *Lam* inactivation has a different effect on *gypsy* regulation in the two genetic backgrounds analyzed. In fact, comparing the *gypsy* expression in somatic adult tissues of *Lam^4643/K2^* with that of *Lam^+/+^*, we found that *gypsy* transcript levels increase in the wild type genetic background while decreasing in that of *flam^A^* (Figure 2E,F). Repression of *gypsy* in *Lam* mutant flies with the *flam^A^* genetic background was also confirmed by analyzing the expression of the *gypsy-lacZ* reporter in ovaries (Figure 2C). To evaluate the effect of *Lam* inactivation on the expression of a single and specific *gypsy* element, we analyzed transcription at the boundary between *gypsy* and the neighbor genomic regions. With this aim, we performed qRT-PCR experiments using specific primers designed to amplify sequences from the flanking genomic sequences into the specific *gypsy* sequences in *flam^A^* flies carrying the *ct^A^* allele, finding a significant downregulation (Figure 2G). *Lam* inactivation has no effect on the transcription found in the same genomic region in absence of the *gypsy* insertion that produces the *ct^A^* allele (Figure S1B), suggesting that the silencing effect depends on the presence of the *gypsy* sequences. These data also suggest that *gypsy* sequences are silenced even when they are part of fused transcripts. This was confirmed by analyzing the expression levels of two *gypsy* fragments that are part of the *flamenco* transcripts. *flamenco* contains fragments of retrotransposon sequences, and the processing of its transcripts produces piRNAs and endo-siRNAs that specifically silence *gypsy* and other TEs [32,33]. Both *gypsy* fragments in the *flamenco* transcript appeared slightly but significantly downregulated (Figure 2H), supporting the hypothesis that *gypsy* sequences are silenced in different parts of the genome, even if they are only portions of the retrotransposon sequence. Furthermore, by adding to the same genetic background a copy of the *ct^A^* allele, the increase of *gypsy* copy number induces a more pronounced silencing effect (Figure 2H). A correlation between cosuppression of *gypsy* sequences, located both in an active *gypsy* element and inside the *flamenco* cluster, and the increase of mortality rate of adult pharate has been previously described [31]. This supports the idea of the involvement of *gypsy* in the induction of the mortality phenotype in *Lam* mutants during the pharate stage.

### 3.2. Silencing of TEs Located Near Euchromatic Genes in Lam Mutant Somatic Tissues

**Figure 3:**
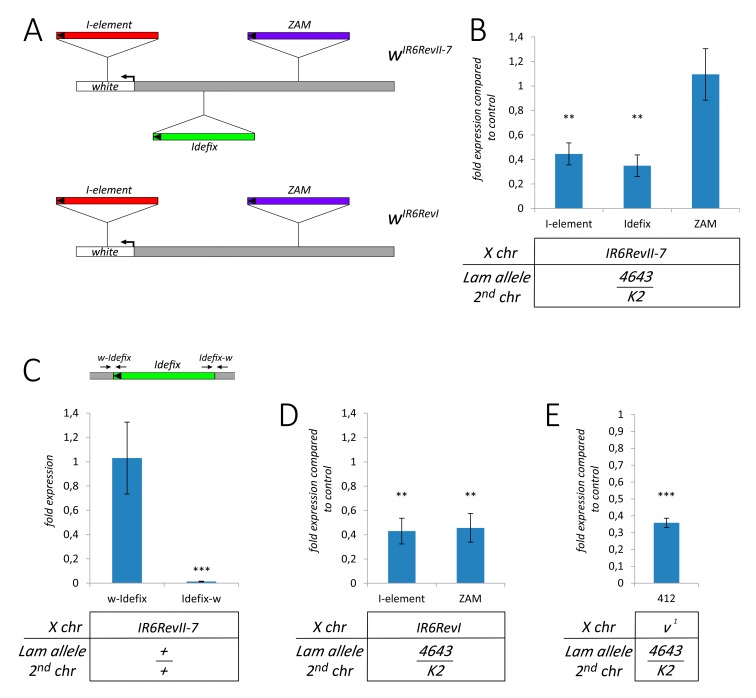
Silencing of euchromatic retrotransposons in somatic tissues of *Lam* mutant flies. (**A**) Schematic representation of the structure of the *w^IR6RevII7^* and *w^IR6RevI^* alleles. The *I-element* (5.4 kb in length) is located in the first intron of the *w* gene. The *Idefix* element (7.4 kb in length) is located at about 1660 bp from the *w* transcription start site. The ZAM element (9 kb in length) is located at about 3 kb from the *w* transcription start site in the *w^IR6RevI^* allele, and more than 10 kb in the *w^IR6RevII7^* alleles. Transcription orientation is indicated by arrows. (**B**–**E**) qRT-PCR analysis of some specific retrotransposons in RNAs isolated from female head tissues. Data are mean values from three independent experiments, and error bars indicate SD (** *P* < 0.01; *** *P* < 0.005). (**B**) *I-element*, *Idefix*, and *ZAM* expression in *Lam^4643^/Lam^K2^* mutants (4643/K2) compared to control flies (+/+) in the *w^IR6RevII7^* mutant background. (**C**) Upper part: schematic representation of the genomic region containing the 5′ upper region of the *white* gene (grey) where the *Idefix* element (green) is inserted. The two qPCR couples of primers are represented by black arrows (see Table S1). Lower part: expression of the regions containing the boundaries between *Idefix* and the *white* 5′ regions in *w^IR6RevII7^* mutants. w-Idefix is the amplicon containing the boundary between the *white* 5′ untranscribed regions and *Idefix*, while Idefix-w is the amplicon containing the boundary between *Idefix* and the genomic region that separates *Idefix* from *ZAM*. (**D**) *I-element* and *ZAM* expression in *Lam^4643/K2^* mutants (4643/K2) compared to control flies (+/+) in the *w^IR6RevI^* mutant background. (**E**) Expression of the *412* retrotransposon in *Lam^04643/K2^* mutants compared to the *Lam^+/+^* in the *v^1^* mutant background.

Inactivation of the *Lam* gene in the fat body of *Drosophila* larvae and adults induces the activation of a number of TEs [25]. A similar activation was observed comparing the expression of TEs between young and old flies, and this was attributed to a reduction of the Lam protein during aging. Interestingly, aging also leads to a silencing of similar number of TEs [25]. Our data suggested that *gypsy* active elements are silenced in *Lam* mutant somatic tissues, so we decided to test the hypothesis that TEs located in a euchromatic active locus can be downregulated when the *Lam* gene is mutated. With this aim, we analyzed the expression of the three TEs in the *white* (*w*) locus of the *w^IR6RevII7^* mutant [34] in adult head tissues. In this mutant, an *I-element* is located inside the first intron of the *white* gene, an *Idefix* element is inserted in the 5′ upstream region at about 1.5 kb from the transcription start site, and a *ZAM* element is located further away from the *white* 5′ region (Figure 3A). In the *Lam^4643/K2^* genetic background, the two TEs closer to the 5′ region of the *white* gene, namely the *I*-*element* and *Idefix*, were significantly downregulated, while *ZAM* expression was not significantly changed (Figure 3B). These data suggested that the expression of the two closer TEs could be influenced by their proximity to the 5′ region of the *white* gene. Due to the presence of *Idefix* between *white* and *ZAM* in the *w^IR6RevII7^* mutant, the *ZAM* element is at about 10 kb from the *white* transcription start site (Figure 3A), and this distance and/or the presence of an insulator inside the *Idefix* sequence [34] could explain the lack of silencing of *ZAM*. We analyzed whether the transcription levels were different comparing the expression of the region containing the boundary between *Idefix* and the *white* untranscribed region and the boundary between *Idefix* and the genomic region that separates *Idefix* from *ZAM* (Figure 3C, upper part). In a *Lam^+/+^* genetic background, we observed a much higher expression at the boundary between *Idefix* and the *white* untranscribed region (Figure 3C, lower part). This finding suggests that *Idefix* and *ZAM* have different genomic environments and that the presence of the *Idefix* element between *ZAM* and the *white* gene in *w^IR6RevII7^* flies is the cause of the insensitivity of *ZAM* expression to the inactivation of *Lam*. To check if the presence of the *Idefix* element between *ZAM* and the *white* gene is the reason why *ZAM* expression is unaffected, we analyzed the expression of this TE in *w^IR6RevI^; Lam^4643/K2^* flies. The *w^IR6RevII7^* allele derives from a de novo integration of a *Idefix* element in the *w^IR6RevI^* allele, which only carries the *I-element* and the *ZAM* insertion in the *white* locus [34] (Figure 3A). In *w^IR6RevI^; Lam^4643/K2^* adult heads, expression of the *I-element* is downregulated as well as the expression of *ZAM* in respect to the control (Figure 3D). Therefore, when *Lam* is inactivated, the absence of the *Idefix* element enables the silencing of the *ZAM* element located upstream of the *white* gene. To confirm the hypothesis of the repression of TEs located near active genes in euchromatic regions as a consequence of *Lam* inactivation, we analyzed the expression level of the *412* element in the *vermilion^1^* (*v^1^*) genetic background. This hypomorphic allele is produced by the insertion of a *412* element in the 5′ UTR region of the *vermilion* gene [35]. We found a significant downregulation comparing *412* expression between the *Lam^4643/K2^* mutant and its respective control (Figure 3E), while we did not find a similar downregulation of the *412* retrotransposon in *Lam^4643/K2^* flies with a wild type genetic background (Figure S2A).

All these data are in agreement with the hypothesis that the silencing of TEs in the presence of non-functional Lam protein could depend on their localization in proximity of active genes in euchromatic regions.

### 3.3. The Effect of Lam Inactivation on TE Expression is Dependent on the Genetic Background

**Figure 4:**
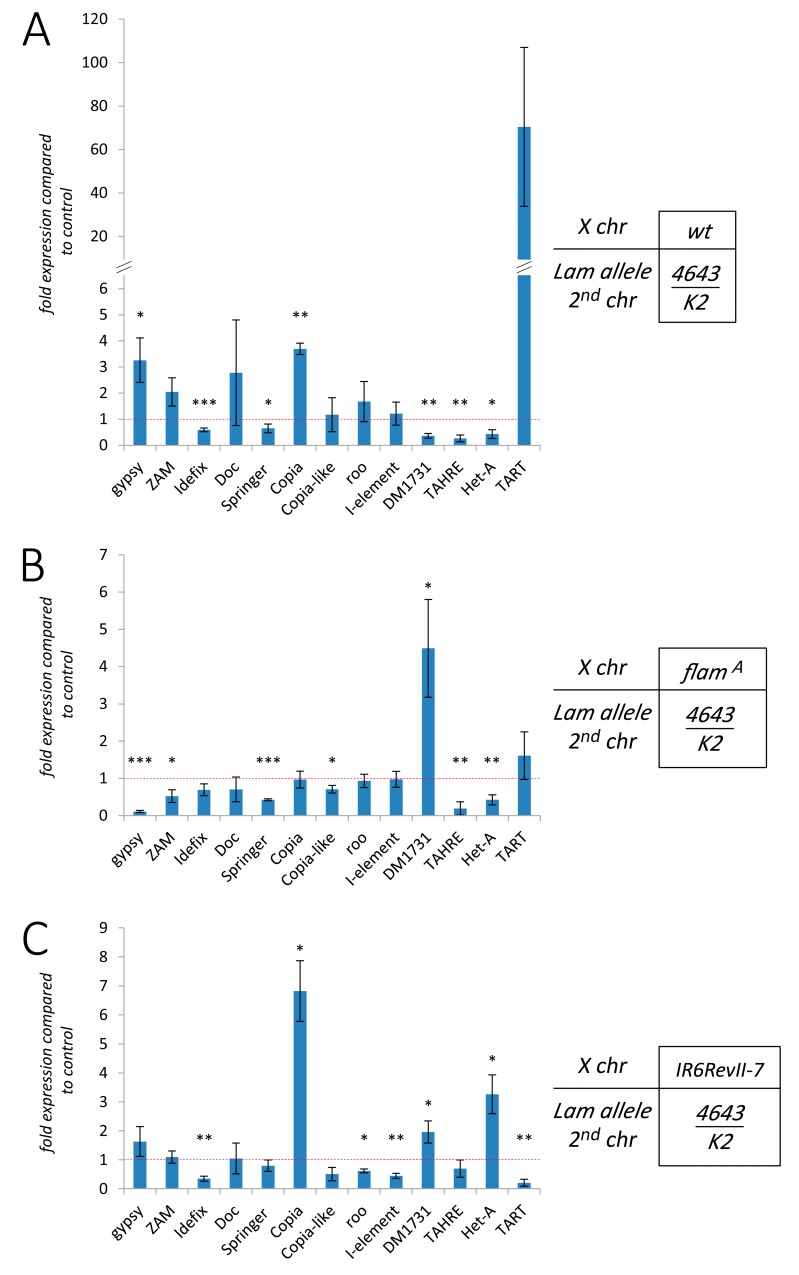
Expression variations of families of TEs in somatic tissues of *Lam* mutants with different genetic backgrounds. (**A**–**C**) qRT-PCR analysis of a number of retrotransposons in RNAs isolated from female head tissues. Histograms indicate the expression level of each TE in *Lam* mutants (4643/K2) compared to that of the specific control (+/+). Data are mean values from three independent experiments, and error bars indicate SD (* *P* < 0.05; ** *P* < 0.01; *** *P* < 0.005). Expression levels in: Canton S genetic background (**A**); *flam^A^* genetic background (**B**); *w^IR6RevII7^* genetic background (**C**).

Our findings suggest that the presence of a TE in a transcriptionally active region can promote the silencing of that TE when the *Lam* gene is mutated. Since the distribution pattern of each TE is different in different genetic backgrounds, we expected that *Lam* mutations could elicit significantly different TE expression levels, depending on the genetic background. To confirm this hypothesis, we analyzed the expression pattern of 13 TEs in three different *Drosophila* strains. In addition to the TEs already analyzed, we selected other LTR and non-LTR retrotransposons, including telomeric retrotransposons. Analysis of the expression of the TEs in the Canton S genetic background comparing *Lam^4643/K2^* with *Lam^+/+^* flies shows that some are upregulated, others are downregulated, while few of them do not show significant differences (Figure 4A). The same samples, which derive from somatic tissues of adult females, show upregulation of three testis-specific genes (Figure S3A). A previous report showed that the most of testis-specific genes interact with Lam in female somatic tissues, and that ablation of *Lam* leads to detachment of these genes from the NL with their consequent transcriptional up-regulation [36]. Our data suggest that some TEs could behave as these repressed genes, while others have a different regulation. Then, comparing the expression of the different TEs in the three different genetic backgrounds, we found a great variability in the expression levels of most of them (Figure 4A–C). These data are in agreement with the hypothesis that the up- or down-regulation of TEs in *Lam* mutant cells depends on their localization. Finally, it is also interesting to note that comparing the expression of the selected TEs in the whole body, we found that some TEs are differently expressed in the head or ovary, suggesting differences in the regulation of TEs depending on the tissues analyzed (Figure S3B). This is not unexpected because some regions of the genome have a different regulation in different tissues.

Our results suggest that NL dysfunction influences TE expression with variable effects depending on the distribution pattern of each mobile element in the genome.

### 3.4. The Silencing of Euchromatic TEs Induced by Lam Inactivation Spreads to Neighbor Genes

**Figure 5:**
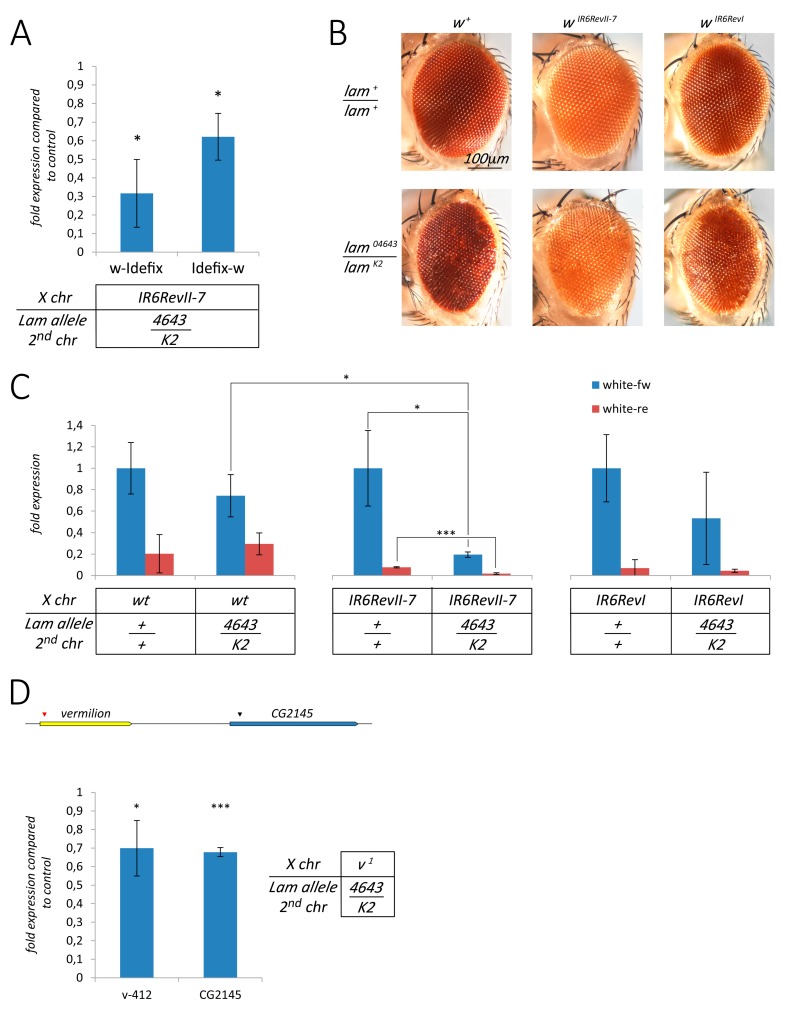
Silencing of the genes located in the neighborhood of silenced euchromatic retrotransposons in *Lam* mutant flies. (**A**) qRT-PCR analysis of the regions containing the boundaries between *Idefix* and the *white* untranscribed regions of *w^IR6RevII7^*; *Lam^04643/K2^* compared to *w^IR6RevII7^*; *Lam^+^*. Data are mean values from three independent experiments, and error bars indicate SD (* *P* < 0.05). (**B**) Representative bright-field microscope images of adult eyes of wild type, *w^IR6RevII7^*, and *w^IR6RevI^* flies with and without *lam* loss-of-function mutant allelic combination. (**C**) qRT-PCR analysis of *white* expression in RNAs isolated from female head tissues. Blue histograms represent sense expression, while red histograms represent anti-sense expression. *white* expression in each genetic background is referred to the sense expression of its specific control. Data are mean values from three independent experiments, and error bars indicate SD (* *P* < 0.05; *** *P* < 0.001). (**D**) Upper part: schematic representation of the genomic region containing the *vermilion* and *CG2145* genes. Black arrowhead indicates the *412* insertion point where v-412 primers were designed, while red arrowhead indicates the region where CG2145 primers were designed (see Table S1). Lower part: qRT-PCR analysis in RNAs isolated from female head tissues in *Lam^04643/K2^* mutants compared to the *Lam^+/+^* in the *v^1^*mutant background. Data are mean values from three independent experiments, and error bars indicate SD (* *P* < 0.05; *** *P* < 0.005).

Silencing of the boundaries between the genomic regulatory regions of the *cut* locus and the *gypsy* element that produces the *ct^A^* allele (Figure 2G) suggested that the silencing of a TE induced by NL dysfunction has the potential to affect the expression of nearby genes. To evaluate a spreading of the silencing from the TE sequences to the flanking genomic regions, we compared the expression of the boundary between *Idefix* and the *white* regulatory regions of *w^IR6RevII7^; Lam^4643/K2^* flies with that of *w^IR6RevII7^; Lam^+/+^* control flies. There was a significant reduction of the expression at both boundaries, more pronounced in the boundary near the *white* gene (Figure 5A). To explore a possible effect of silencing on the *white* gene, we compared the eye color of *Lam^4643/K2^* in the wild type and in the two *white* mutant backgrounds with that of relative controls (Figure 5B). In the wild type background, we found a reduction of eye pigmentation in some regions of the eye (Figure 5B) that could depend on the eye alteration induced by the *Lam* mutations [30] or on a low silencing effect. Analysis of the expression inside the coding region of the *white* gene, in sense and antisense direction, showed a small (not significant) reduction (Figure 5C). We obtained similar results by qRT-PCR experiments performed using random primers, which allow the analysis of the whole transcript level (Figure S4). A reduction of the eye pigmentation was clearly found in *w^IR6RevII7^* and *w^IR6RevI^* files that carried the *Lam^4643/K2^* combination in respect to the controls (Figure 5B). Inactivation of *Lam* induces a significant silencing of *white* expression in the *w^IR6RevI^* and *w^IR6RevII7^* genetic background in respect to their controls (Figure 5C and Figure S4). The *white* silencing in the *w^IR6RevII7^* genetic background appears more effective and was significantly higher than in the wild type genetic background (Figure 5C and Figure S4). This supports the hypothesis that the effect is induced by the presence of the TE sequences near there and that the *Idefix* element induces the strongest effect of silencing on the *white* gene.

To confirm these data, we analyzed whether the insertion of the *412* element in the *vermilion* locus has a silencing effect on the surrounding genomic regions. As expected, we found that the insertion of the *412* element in the *vermilion* locus determines a strong reduction in the expression of the *vermilion* gene (Figure S2B). To analyze a possible silencing effect on the genomic region containing the *vermilion* gene due to a *Lam* loss-of-function, we compared the expression level of the *CG2145* gene in *Lam^4643/K2^* mutants with that of the respective controls in the wild type genetic background. The *CG2145* gene, which is located downstream of the *vermilion* gene at about 3.5 kb, is not downregulated by *Lam* inactivation in a wild type genetic background (Figure S2C). However, in the *v^1^* genetic background, inactivation of *Lam* produces a significant downregulation of the boundary between the *412* element and the *vermilion* gene and of the *CG2145* gene (Figure 5D). This confirms the hypothesis that the inactivation of *Lam* induces the silencing of TEs located in euchromatic genes, which has the potential to spread into the flanking regions near the TE insertion site.

## 4. Discussion

**Figure 6:**
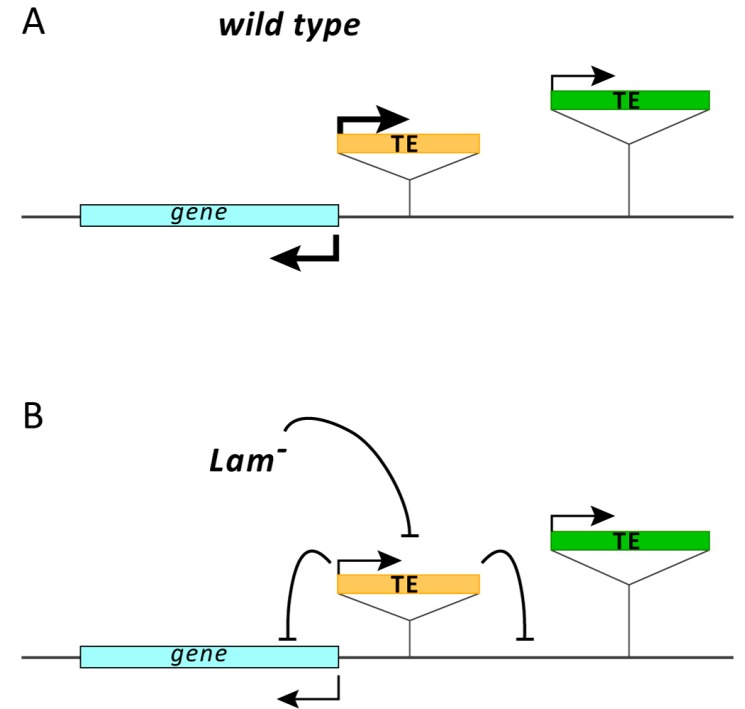
A model of the *Lamin* inactivation effects on euchromatic TEs and the neighboring genomic regions. (**A**) In wild type strains, TEs inserted near expressed genes are normally active due to the open structure of euchromatin that is permissive for transcription. (**B**) When the Lamin is inactivated, TEs close to active genes are silenced, while those located in less active regions are not affected. The silencing effect on euchromatic TEs spreads to the neighboring genomic regions affecting the expression of nearby genes. The thickness of arrows indicates the levels of expression.

In *Drosophila*, derepression of TEs has been correlated with a reduction of Lamin protein caused by aging or by mutations affecting the *Lam* gene [25]. It has been found that the increase in the expression of TEs is associated with a reduction of heterochromatin at TE sequences, providing a convincing explanation of the cause of derepression. In human and mouse, an enrichment of *LINE* elements has been found at lamina associated domains (LADs), heterochromatic regions that are found at the nuclear periphery [37,38]. *LINE-1* is derepressed during senescence [39] or as a consequence of *LMNA* silencing and when the H3K18 deacetylation is inhibited by mutation of *SIRT7*, which reduces association between *LINE-1* sequences and lamin A/C [26]. Less obvious is the mechanism that leads to the silencing of some TEs in flies with reduced or mutated *Lamin*. Presence of repressed TEs was found comparing the expression levels between old and young flies [25], and, in the present study, where the analysis was performed comparing *Lamin* mutants with their controls. A possible explanation could be the different genomic environment in which TEs are located, which would lead to a different regulatory response to NL dysfunction. Here we report that proximity to an active gene could be an important feature in determining the repressive response and, in fact, we found that the *I-element* located in the first intron of the *white* gene and the LTR retrotransposons located in 5′ regulatory regions are repressed when the *Lam* gene is mutated. The open structure of euchromatin is permissive for transcription, and an euchromatic TE should remain expressed and insensitive to NL dysfunction. If other copies of the same TE are present in heterochromatic regions, there should be an increase in the overall expression level. However, in a previous study we demonstrated that new euchromatic insertions of the retrotransposon *gypsy* in a *Lam^+^* genetic background causes the silencing of this retrotransposon despite the increase in copy number that theoretically should increase the expression level [31]. This happens without increase of H3K9me3 and H3K27me3 at *gypsy* sequences, suggesting a post-transcriptional silencing. Furthermore, *gypsy* homologous sequences found in RNA precursors of sncRNAs that silence *gypsy* and other TEs also appeared downregulated, supporting a cosuppression model. Cosuppression is a form of post-transcriptional gene silencing first reported in transgenic petunia, where a transgene inserted to overexpress the host Chalcone Synthase-A gene gave rise to degradation of the homologous host transcript and the consequent loss of flower pigmentation [40]. A possible explanation of our findings is that NL dysfunction could mimic the effect of the increase of copy number, activating a post-transcriptional RNA silencing of TEs located in transcriptionally active regions. Indeed, a general reduction of TE transcript level due to degradation is compatible with a condition of transcriptional activation [41]. In fact, if a higher level of transcript was associated with an increase of its processing and, consequently, of sncRNAs, this should also increase RNA-mediated silencing. Our study suggests that *Lamin* inactivation has a repressive effect on TEs located nearby euchromatic active genes (Figure 6). A consequence of this effect is the silencing spreading to the flanking genomic regions. We found that this silencing can extend up to 3.5 kb when *412* is silenced by *Lam* inactivation. Spreading of repressive epigenetic marks that are directed to silence TEs and that can repress transcription of neighboring genes is a known phenomenon [42,43,44]. In fact, it is reported that this occurs in more than 50% of the euchromatic TEs and can extend up to 20 kb in the flanking genomic regions [43]. A study performed on *Drosophila* ovaries demonstrates that newly transposed euchromatic TEs become piRNAs and endo-siRNAs clusters and that the production of sncRNAs spreads into TE flanking genomic regions, which can change the expression of nearby genes [45]. Production of endo-siRNAs from TE flanking genomic regions is also compatible with a post-transcriptional RNA silencing. Although further studies will be necessary to understand the mechanism that induces the silencing of some TEs in *Lamin* mutant flies and during the aging process, a posttranscriptional silencing mechanism appears conceivable. 

A previous study shows how cosuppression of *gypsy* and homologous sequences is associated with an increase of mortality during adult eclosion [31]. Mortality during the eclosion of the adult stage has also been reported as one of the major periods of lethality of *Lamin* mutants [22]. By increasing the number of *gypsy* insertions in a genetic background showing low level of mortality during adult eclosion, we assisted an increase of this lethal phenotype induced by *Lamin* inactivation (Figure 1 and Figure S1A). These findings support the hypothesis that this phenotype could be linked to *gypsy* silencing, opening the intriguing possibility that silencing of TEs triggered by NL dysfunctions could also be involved in some features of laminopathic disease. Silencing of TEs can spread to the flanking genomic regions [43], and we found a similar effect on genes located near euchromatic TEs as a consequence of *Lamin* mutation (Figure 6). This effect could affect a number of genes located in the neighborhood of euchromatic TEs with possible implications for one or more phenotypes. Investigation of the effects of *LMNA* mutations on the expression of human TEs could be useful to understand a possible role of TEs in laminopathies and could help to find an explanation to the high phenotype variability observed in muscular laminopathies [46]. In fact, a high variability in phenotype severity, also within the same family, makes it challenging to establish a genotype-phenotype correlation. This suggests a prominent role of the genetic background. In this regard, the extreme variability in the patterns of the distribution of TEs in genomes could be an important determinant of phenotypic variability.

## Supplementary Materials

The following are available online at https://www.mdpi.com/2073-4409/9/3/625/s1, Figure S1: The *gypsy* insertion in the *forked* (*f*) locus increases the mortality rate induced by *Lam* inactivation; Figure S2: *Lam* inactivation does not affect the expression of the *412* retrotransposon element, of *vermilion* (*v*) and *CG2155* genes in the wild type genetic background; Figure S3: Expression of testis specific genes in the wild type genetic background and of TEs in whole females, somatic tissues, and ovaries; Figure S4: Silencing of the *white* gene is induced by TEs located in the 5’ untranscribed region; Table S1: Primer sequences used in this study; Table S2: Phenotypes of *Lam* mutants.

## References

[1] Bourque G., Burns K.H., Gehring M., Gorbunova V., Seluanov A., Hammell M., Imbeault M., Izsvak Z., Levin H.L., Macfarlan T.S. (2018). Ten things you should know about transposable elements. Genome Biol..

[2] Nefedova L., Kim A. (2017). Mechanisms of LTR-Retroelement Transposition: Lessons from Drosophila melanogaster. Viruses.

[3] Han J.S. (2010). Non-long terminal repeat (non-LTR) retrotransposons: Mechanisms, recent developments, and unanswered questions. Mob. DNA.

[4] Buchon N., Vaury C. (2006). RNAi: A defensive RNA-silencing against viruses and transposable elements. Heredity.

[5] Castel S.E., Martienssen R.A. (2013). RNA interference in the nucleus: Roles for small RNAs in transcription, epigenetics and beyond. Nat. Rev. Genet..

[6] Driver C.J., McKechnie S.W. (1992). Transposable elements as a factor in the aging of Drosophila melanogaster. Ann. N. Y. Acad. Sci..

[7] Shevelyov Y.Y., Ulianov S.V. (2019). The Nuclear Lamina as an Organizer of Chromosome Architecture. Cells Basel.

[8] De Leeuw R., Gruenbaum Y., Medalia O. (2018). Nuclear Lamins: Thin Filaments with Major Functions. Trends Cell Biol..

[9] Adam S.A., Goldman R.D. (2012). Insights into the differences between the A- and B-type nuclear lamins. Adv. Biol. Regul..

[10] Camozzi D., Capanni C., Cenni V., Mattioli E., Columbaro M., Squarzoni S., Lattanzi G. (2014). Diverse lamin-dependent mechanisms interact to control chromatin dynamics. Focus on laminopathies. Nucl. Phila..

[11] Cenni V., D’Apice M.R., Garagnani P., Columbaro M., Novelli G., Franceschi C., Lattanzi G. (2018). Mandibuloacral dysplasia: A premature ageing disease with aspects of physiological ageing. Ageing Res. Rev..

[12] Ditaranto R., Boriani G., Biffi M., Lorenzini M., Graziosi M., Ziacchi M., Pasquale F., Vitale G., Berardini A., Rinaldi R. (2019). Differences in cardiac phenotype and natural history of laminopathies with and without neuromuscular onset. Orphanet J. Rare Dis..

[13] Mattioli E., Columbaro M., Capanni C., Maraldi N.M., Cenni V., Scotlandi K., Marino M.T., Merlini L., Squarzoni S., Lattanzi G. (2011). Prelamin A-mediated recruitment of SUN1 to the nuclear envelope directs nuclear positioning in human muscle. Cell Death Differ..

[14] Pellegrini C., Columbaro M., Schena E., Prencipe S., Andrenacci D., Iozzo P., Angela Guzzardi M., Capanni C., Mattioli E., Loi M. (2019). Altered adipocyte differentiation and unbalanced autophagy in type 2 Familial Partial Lipodystrophy: An in vitro and in vivo study of adipose tissue browning. Exp. Mol. Med..

[15] Bohnekamp J., Cryderman D.E., Thiemann D.A., Magin T.M., Wallrath L.L. (2016). Using Drosophila for Studies of Intermediate Filaments. Methods Enzymol..

[16] Bossie C.A., Sanders M.M. (1993). A Cdna from Drosophila-Melanogaster Encodes a Lamin C-Like Intermediate Filament Protein. J. Cell Sci..

[17] Palka M., Tomczak A., Grabowska K., Machowska M., Piekarowicz K., Rzepecka D., Rzepecki R. (2018). Laminopathies: What can humans learn from fruit flies. Cell. Mol. Biol. Lett..

[18] Riemer D., Weber K. (1994). The Organization of the Gene for Drosophila Lamin-C—Limited Homology with Vertebrate Lamin Genes and Lack of Homology Versus the Drosophila Lamin Dmo Gene. Eur. J. Cell Biol..

[19] Smith D.E., Gruenbaum Y., Berrios M., Fisher P.A. (1987). Biosynthesis and interconversion of Drosophila nuclear lamin isoforms during normal growth and in response to heat shock. J. Cell Biol..

[20] Riemer D., Stuurman N., Berrios M., Hunter C., Fisher P.A., Weber K. (1995). Expression of Drosophila Lamin-C Is Developmentally-Regulated -Analogies with Vertebrate a-Type Lamins. J. Cell Sci..

[21] Munoz-Alarcon A., Pavlovic M., Wismar J., Schmitt B., Eriksson M., Kylsten P., Dushay M.S. (2007). Characterization of lamin Mutation Phenotypes in Drosophila and Comparison to Human Laminopathies. PLoS ONE.

[22] Lenz-Bohme B., Wismar J., Fuchs S., Reifegerste R., Buchner E., Betz H., Schmitt B. (1997). Insertional mutation of the Drosophila nuclear lamin Dm0 gene results in defective nuclear envelopes, clustering of nuclear pore complexes, and accumulation of annulate lamellae. J. Cell Biol..

[23] Worman H.J., Bonne G. (2007). “Laminopathies”: A wide spectrum of human diseases. Exp. Cell Res..

[24] Osouda S., Nakamura Y., de Saint Phalle B., McConnell M., Horigome T., Sugiyama S., Fisher P.A., Furukawa K. (2005). Null mutants of Drosophila B-type lamin Dm(0) show aberrant tissue differentiation rather than obvious nuclear shape distortion or specific defects during cell proliferation. Dev. Biol..

[25] Chen H., Zheng X., Xiao D., Zheng Y. (2016). Age-associated de-repression of retrotransposons in the Drosophila fat body, its potential cause and consequence. Aging Cell.

[26] Vazquez B.N., Thackray J.K., Simonet N.G., Chahar S., Kane-Goldsmith N., Newkirk S.J., Lee S., Xing J., Verzi M.P., An W. (2019). SIRT7 mediates L1 elements transcriptional repression and their association with the nuclear lamina. Nucleic Acids Res..

[27] Pelisson A., Mejlumian L., Robert V., Terzian C., Bucheton A. (2002). Drosophila germline invasion by the endogenous retrovirus gypsy: Involvement of the viral env gene. Insect Biochem. Mol. Biol..

[28] Li W., Prazak L., Chatterjee N., Gruninger S., Krug L., Theodorou D., Dubnau J. (2013). Activation of transposable elements during aging and neuronal decline in Drosophila. Nat. Neurosci..

[29] Guillemin K., Williams T., Krasnow M.A. (2001). A nuclear lamin is required for cytoplasmic organization and egg polarity in Drosophila. Nat. Cell Biol..

[30] Patterson K., Molofsky A.B., Robinson C., Acosta S., Cater C., Fischer J.A. (2004). The functions of Klarsicht and nuclear lamin in developmentally regulated nuclear migrations of photoreceptor cells in the Drosophila eye. Mol. Biol. Cell.

[31] Guida V., Cernilogar F.M., Filograna A., De Gregorio R., Ishizu H., Siomi M.C., Schotta G., Bellenchi G.C., Andrenacci D. (2016). Production of Small Noncoding RNAs from the flamenco Locus Is Regulated by the gypsy Retrotransposon of Drosophila melanogaster. Genetics.

[32] Brennecke J., Aravin A.A., Stark A., Dus M., Kellis M., Sachidanandam R., Hannon G.J. (2007). Discrete small RNA-generating loci as master regulators of transposon activity in Drosophila. Cell.

[33] Ghildiyal M., Seitz H., Horwich M.D., Li C., Du T., Lee S., Xu J., Kittler E.L., Zapp M.L., Weng Z. (2008). Endogenous siRNAs derived from transposons and mRNAs in Drosophila somatic cells. Science.

[34] Desset S., Vaury C. (2005). Transcriptional interference mediated by retrotransposons within the genome of their host: Lessons from alleles of the white gene from Drosophila melanogaster. Cytogenet. Genome Res..

[35] Searles L.L., Ruth R.S., Pret A.M., Fridell R.A., Ali A.J. (1990). Structure and transcription of the Drosophila melanogaster vermilion gene and several mutant alleles. Mol. Cell. Biol..

[36] Shevelyov Y.Y., Lavrov S.A., Mikhaylova L.M., Nurminsky I.D., Kulathinal R.J., Egorova K.S., Rozovsky Y.M., Nurminsky D.I. (2009). The B-type lamin is required for somatic repression of testis-specific gene clusters. Proc. Natl. Acad. Sci. USA.

[37] Meuleman W., Peric-Hupkes D., Kind J., Beaudry J.B., Pagie L., Kellis M., Reinders M., Wessels L., van Steensel B. (2013). Constitutive nuclear lamina-genome interactions are highly conserved and associated with A/T-rich sequence. Genome Res..

[38] Zullo J.M., Demarco I.A., Pique-Regi R., Gaffney D.J., Epstein C.B., Spooner C.J., Luperchio T.R., Bernstein B.E., Pritchard J.K., Reddy K.L. (2012). DNA Sequence-Dependent Compartmentalization and Silencing of Chromatin at the Nuclear Lamina. Cell.

[39] De Cecco M., Ito T., Petrashen A.P., Elias A.E., Skvir N.J., Criscione S.W., Caligiana A., Brocculi G., Adney E.M., Boeke J.D. (2019). L1 drives IFN in senescent cells and promotes age-associated inflammation. Nature.

[40] Jorgensen R.A. (1995). Cosuppression, flower color patterns, and metastable gene expression States. Science.

[41] Andrenacci D., Cavaliere V., Lattanzi G. (2020). The role of transposable elements activity in aging and their possible involvement in laminopathic diseases. Ageing Res. Rev..

[42] Lee Y.C. (2015). The Role of piRNA-Mediated Epigenetic Silencing in the Population Dynamics of Transposable Elements in Drosophila melanogaster. PLoS Genet..

[43] Lee Y.C.G., Karpen G.H. (2017). Pervasive epigenetic effects of Drosophila euchromatic transposable elements impact their evolution. eLife.

[44] Sienski G., Donertas D., Brennecke J. (2012). Transcriptional silencing of transposons by Piwi and maelstrom and its impact on chromatin state and gene expression. Cell.

[45] Shpiz S., Ryazansky S., Olovnikov I., Abramov Y., Kalmykova A. (2014). Euchromatic Transposon Insertions Trigger Production of Novel Pi- and Endo-siRNAs at the Target Sites in the Drosophila Germline. PLoS Genet..

[46] Maggi L., Carboni N., Bernasconi P. (2016). Skeletal Muscle Laminopathies: A Review of Clinical and Molecular Features. Cells (Basel).

