# Supplementary material for: Silencing of Euchromatic Transposable Elements as a Consequence of Nuclear Lamina Dysfunction
Source: Cells. 2020 Mar 5;9(3):625. doi: 10.3390/cells9030625 (PMC7140440; doi:10.3390/cells9030625)
Supplement: Supplementary file 1 [file cells-09-00625-s001.pdf]

cells MDPI

Supplementary Materials:

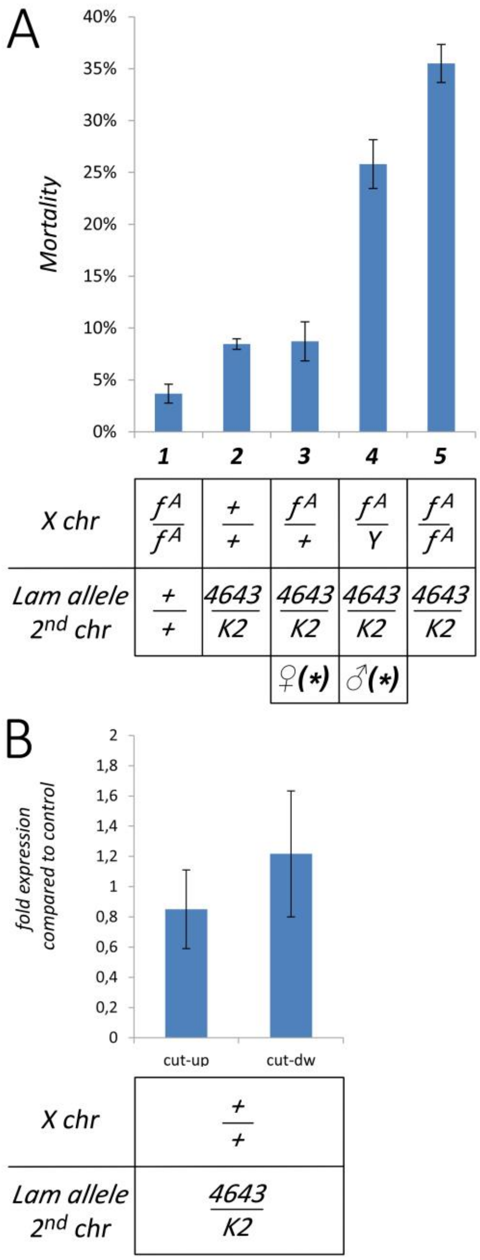

Figure S1. The *gypsy* insertion in the *forked* (*f*) locus increases the mortality rate induced by *Lam* inactivation. (**A**) Mortality during adult eclosion of pharate with different genotypes. Columns 1–5: flies with X chromosomes carrying the *flamenco* permissive allele *flam*^{A}, which allows *gypsy* activation. These flies can have the *gypsy* induced mutation (*f*^{A}) in heterozygosis, in homozygosis/hemizygosis or the wild type allele (+). Flies can be homozygous for the wild type *Lam* allele (+/+) or transheterozygous (4643/K2). Y: Y chromosome. Asterisk: females and males derived from the same genetic cross. (**B**) Strand-specific qRT-PCR analysis of the transcription levels in the genomic regions containing the *gypsy* insertion site of the *ct*^{A} allele in *w*$^{+}$; *Lam*$^{4643/K2}$ female head tissues compared to the *w*$^{+}$; *Lam*$^{+}$ control. The two strand-specific primers used for RT experiments were the same of Fig. 1G, while the two qPCR couples of primers were designed in *cut* genomic regions (see tab. S1).

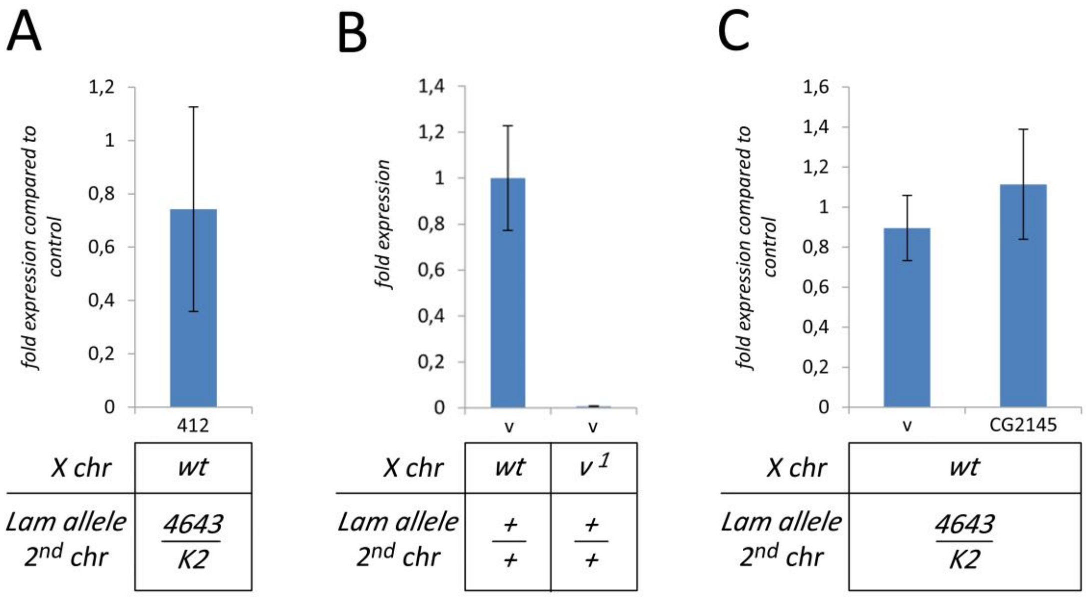

Figure S2. *Lam* inactivation does not affect the expression of the *412* retrotransposon element, of *vermilion* (*v*) and *CG2155* genes in the wild type genetic background. (**A**–**C**) qRT-PCR analysis of RNAs isolated from female head tissues. Data are mean values from three independent experiments, and error bars indicate SD. (**A**) Expression level of the *412* element in $Lam^{4643/K2}$ mutants (4643/K2) compared to control flies (+/+) in the wild type genetic background. (**B**) Effect of the mutation induced by the *412* element, which produces the v^1 allele on the expression of the *vermilion* gene. (**C**) Expression levels of *vermilion* and *CG2145* in $Lam^{4643/K2}$ mutants (4643/K2) compared to control flies (+/+) in the wild type genetic background.

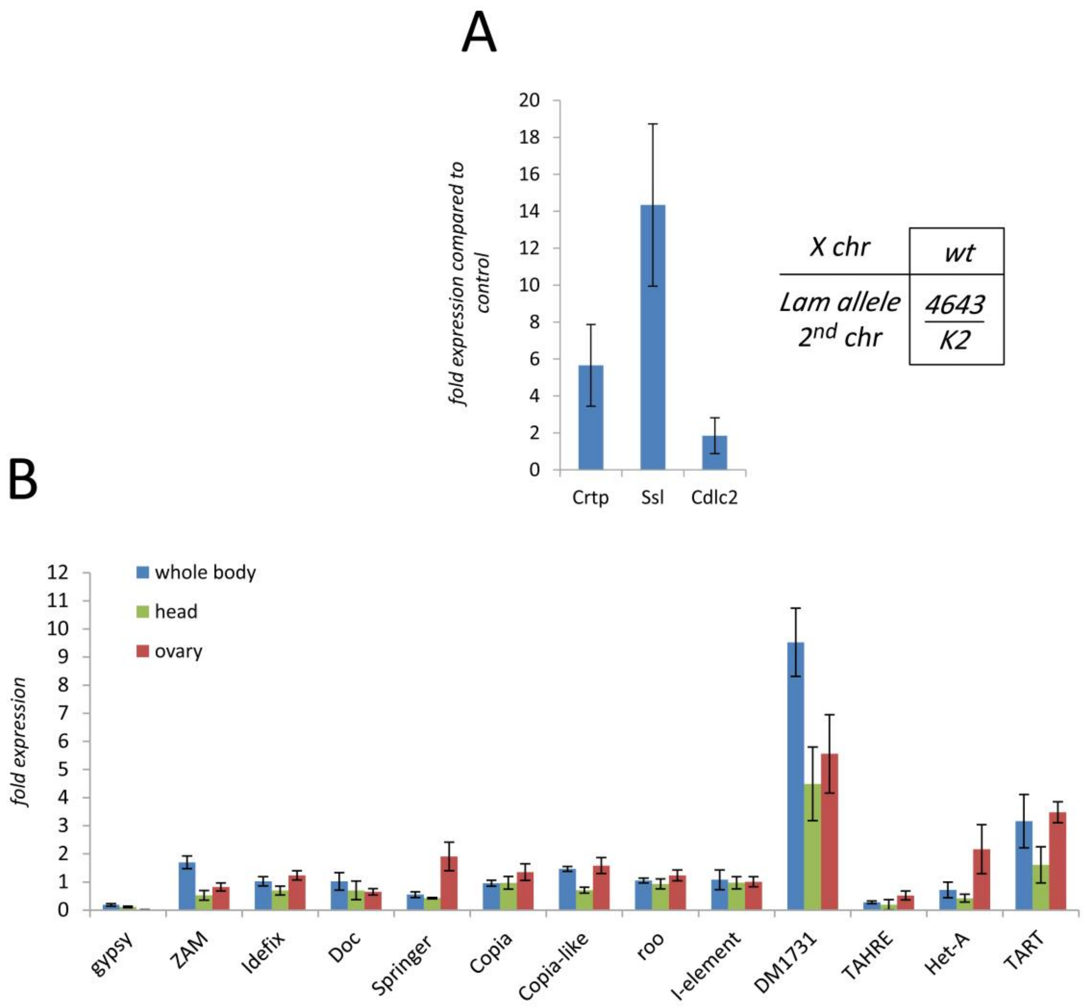

Figure S3. Expression of testis specific genes in the wild type genetic background and of TEs in whole females, somatic tissues, and ovaries. (**A**) qRT-PCR expression analysis of testis specific genes comparing w^+; $Lam^{4643/K2}$ with w^+; Lam^+ somatic tissues. (**B**) Compared expression of the different TEs in $flam^A$; $lam^{4643/K2}$ mutants.

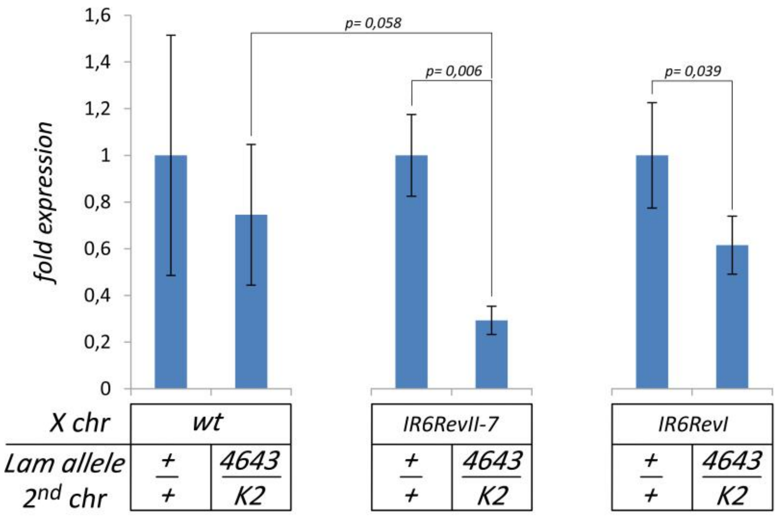

Figure S4. Silencing of the *white* gene is induced by TEs located in the 5′ untranscribed region. qRT-PCR analysis of *white* expression in RNAs isolated from female head tissues of $Lam^{4643/K2}$ mutants (4643/K2) compared to control flies (+/+) in different *white* genetic backgrounds. Data are mean values from three independent experiments, and error bars indicate SD. The p-value has been calculated by one-tail Student's t-test.

Table S1. Primer sequences used in this study

Primers for RT	Forward Primer	Reverse Primer
rp49-RT[1]	5' GACAATCTCCTTGCGCTTCT 3'	
cut-RT up[1]	5' CGGAGAGTTCGGCATTTG 3'	
cut-RT dw[1]		5' CAAGGGGTTGCCTCTCATT 3'
white-fr	5′CAGATGCTCGGCAGATGG3′	
white-re		5′ACACAAAGTGCTGTGCCAAA3′
**Primers for qPCR**		
rp49[1]	5′ TCTGCATGAGCAGGACCTC 3 ′	5′ ATCGGTTACGGATCGAACAA3 ′
gypsy[1]	5′ AGACGCTGCGACCATTCAC 3′	5′ CGTGCTGCCTCCAGAATGAT 3′
cut-gyp up[1]	5′ GGGCTGGGGAATAGAAAACT 3′	5′ TTCATCCCAACTCTTAAAACGAA 3′
cut-gyp dw[1]	5′ ATCCCCCAAAAGGAAGTGAT 3′	5′ AAATGCGCGAAATCTCTCAG 3′
cut-up[1]	5' GGGCTGGGGAATAGAAAACT 3'	5' TTCATCCCAACTCTTAAAACGAA 3'
cut-dw[1]	5' ATCCCCCAAAAGGAAGTGAT 3'	5'AAATGCGCGAAATCTCTCAG 3'
flam1[1]	5′ TCAAAGCGATTCATTCCTCAG 3′	5′ CCATTTGGCTATGAGGATCAG 3′
flam5[1]	5′ CAGGCCCCCTATTGATTAGAT 3′	5′ TGCTCGGGCTTTCTTAAAGT 3′
flam6[1]	5′ GTATATCGGATGGCCGATTG 3′	5′ GCACCGCAAATCATAACGTA 3′
white	5′ CCGCGAATTAATAGCTCCTG 3′	5′ ATTGGGGTGGTGATTGGTT 3′
I-element	5′ ACGAATCGGGTACGAAACAG 3′	5′ TTGCATATGGGTGTTGGATG 3′
ZAM	5′ CTAGACGGGACAGGGAACAG 3′	5′GATGGGGTATCTGTCCGAAA 3′
Idefix[1]	5′ GAATGATTCCGCTCTAGTGG 3′	5′ ATGCGGTCTCTTTCTTCTGC 3′
412	5′ CGCAAAACAGATCAACACAAG 3′	5′ TAGCACACTGTTTGCGTTCC 3′
Doc	5′ TCAGAAACGCACCTCACAAA 3′	5′ GTGCCTCCATGAGCTTTACC 3′
Springer	5′ CTGGAGGAACTCGCCAACAT 3′	5′ CTACGTCGTCCTGGATTAGC 3′
copia[1]	5′ TCTGGTGCTAGTGACCATCT 3′	5′ GCTTGGCCACTGCAATCTTA 3′
copia-like	5′ CTCTACGCTGGACAACCAAT 3′	5′ CTTGTGTCGACTTCGTACTC 3′
roo	5′ CCTCGCAGTAGCGAGTCAGT 3′	5′ GAACGGAGCCCAAAATTGTA 3′
DM1731	5′ GAGAAATCACTTTGGGCCAT 3′	5′ TCGTCGCTGGTCTACAGTTC 3′
TAHRE	5′ ATCCAGGCCAAGGATATGAC 3′	5′ TCTGATGATGACTCGGAAGC 3′
Het-A	5′ ACAGATGCCAAGGCTTCAGG 3′	5′ GCCAGCGCATTTCATGC 3′
TART	5′ TTCCGAGATCCAATCTTCGT 3′	5′ GGGCATCAATATTTAGAATGAACA 3′
w-Idefix	5′ CCGCACAGTCACACCTACAT 3′	5′ AAGGTTCGGTGTTCTTCTCAAA 3′
Idefix-w	5′ ATGGCTGGGGACTTACCTTT 3′	5′ TTGGGTACATCCGGAGTAGTG 3′
v-412	5′ GAGGAGTCACGGGCCTAAC 3′	5′ GGCAGCACTTTGTTGCTATG 3′
CG2145	5′ GCAGTGTTTTTGTGCCTGAC 3′	5′ GCTGGACTTCTTGGTTGTCG 3′
Crtp	5′ CTCCAAGAGAAGGCGGAGA 3′	5′ GTGGCTTCCATAGCGACTGT 3′
Ssl	5′ TGCCAAGCTTGATACCCTCT 3′	5′ ACCTTTATTGGCGGGGACT 3′
Cdlc2	5′ AACAGAGCAAGAGCCGGATA 3′	5′ TGTCAGCGTTCTTGATCACC 3′

Primers design was performed using Primer 3 [1]

[1] [2]

Table S2. Phenotypes of *Lam* mutants

Phenotype		*Lam*$^{4643/+}$	*Lam*$^{4643/4643}$	*Lam*$^{K2/+}$	*Lam*$^{K2/K2}$	*Lam*$^{4643/K2}$
[1]adult-pharate lethality	% (n)	1,0 (311)	63,7 (118)	0,3 (325)	56,3 (109)	8,4 (356)
[2]locomotor defects		-	+	-	+	+
[2]eye defects		-	+	-	+	+
[2]female sterility		-	+	-	+	+
[2]ovary defects		-	+	-	+	+
[2]premature aging		-	+	-	+	+

Phenotypes of *Lam* mutants in the *flam*^{A} genetic background that we used in this study.

[1]Homozygous for *Lam* loss-of-function alleles show higher level of lethality during the adult-pharate stage respect to the *Lam*$^{4643/K2}$ transheterozygous combination. This probably depends on the homozygosity of the second chromosome, which carries the *Lam* locus. To minimize the genetic background effects that derive from homozygous chromosomes, we used the *Lam*$^{4643/K2}$ transheterozygous combination for our genetic analysis. (n): total number of adult-pharate analyzed.

[2](-): flies that do not show the phenotype. (+): flies that show a full penetrant phenotype.

References

1. Untergasser, A.; Cutcutache, I.; Koressaar, T.; Ye, J.; Faircloth, B.C.; Remm, M.; Rozen, S.G. Primer3--new capabilities and interfaces. *Nucleic acids research* **2012**, *40*, e115, doi:10.1093/nar/gks596.
2. Guida, V.; Cernilogar, F.M.; Filograna, A.; De Gregorio, R.; Ishizu, H.; Siomi, M.C.; Schotta, G.; Bellenchi, G.C.; Andrenacci, D. Production of Small Noncoding RNAs from the flamenco Locus Is Regulated by the gypsy Retrotransposon of *Drosophila melanogaster. Genetics* **2016**, *204*, 631-644, doi:10.1534/genetics.116.187922.
